# Pre-natal and early life lead exposure and childhood inhibitory control: an item response theory approach to improve measurement precision of inhibitory control

**DOI:** 10.1186/s12940-023-01015-5

**Published:** 2024-09-05

**Authors:** Shelley H. Liu, Yitong Chen, David Bellinger, Erik de Water, Megan Horton, Martha M. Téllez-Rojo, Robert Wright

**Affiliations:** 1 Department of Population Health Science and Policy, Icahn School of Medicine at Mount Sinai, New York, NY USA; 2 Department of Neurology, Boston Children’s Hospital, Boston, MA USA; 3 Great Lakes Neurobehavioral Center, Edina, MN USA; 4 Department of Environmental Medicine and Public Health, Icahn School of Medicine at Mount Sinai, New York, NY USA; 5 Center for Nutrition and Health Research, National Institute of Public Health, Cuernavaca, Morelos Mexico

**Keywords:** Item response theory, Lead, Neurodevelopment, Inhibitory control, Psychometrics

## Abstract

**Background:**

Neurodevelopmental performance tasks are often separately analyzed, even when they tap into a similar construct. This may yield mixed findings for associations of an exposure-neurobehavioral outcome. We develop an item response theory (IRT) approach to integrate multiple task variables together to improve measurement precision of the underlying construct. We apply this approach to create an integrative measure of childhood inhibitory control, and study impacts of pre/post-natal lead exposure.

**Methods:**

Using data from a prospective cohort based in Mexico (N = 533), we created an inhibitory control scale that integrates accuracy and reaction time information from four inhibitory control tasks (Go/NoGo Letter, Go/NoGo Neutral, Go/NoGo Happy, Delis-Kaplan Executive Function System (D-KEFS) Color-Word Interference Test, Condition 3). Using a generalized partial credit item response theory model, we estimated an inhibitory control index for each participant. We then assessed adjusted associations between umbilical cord blood and 4-year lead and childhood inhibitory control. We developed a resampling approach to incorporate error estimates from the inhibitory control variable to confirm the consistency of the lead-inhibitory control associations. We modeled time-varying associations of lead with each inhibitory control measure separately.

**Results:**

Participants had a median age of 9 years; 51.4% were males. Umbilical cord blood [-0.06 (95% CI: -0.11, -0.01)] and 4-year lead [-0.07 (95% CI: -0.12, -0.02)] were associated with inhibitory control index at 8–10 years. A resampling approach confirmed that 4-year lead was consistently associated with childhood inhibitory control index. Umbilical cord blood and 4-year lead were each associated with 3 out of 8 measures in separate models.

**Conclusion:**

This is the first application of IRT in environmental epidemiology to create a latent variable for inhibitory control that integrates accuracy and reaction time information from multiple, related tasks. This framework can be applied to other correlated neurobehavioral assessments or other phenotype data.

**Supplementary Information:**

The online version contains supplementary material available at 10.1186/s12940-023-01015-5.

## Key messages

- We are among the first to use item response theory to create an integrative measure of childhood inhibitory control, using multiple performance-tasks to improve measurement precision and construct validity.
- We combined information about speed and accuracy on each inhibitory control performance-task using theory driven considerations.
- We developed a resampling approach using plausible value imputation to account for measurement error of inhibitory control factor scores in estimating associations between lead exposures and inhibitory control.
- Higher lead exposure at birth, and during preschool age, were both associated with worse childhood inhibitory control.
- In boys, lead exposure at birth was associated with worse childhood inhibitory control, while in girls, lead exposure during preschool age was associated with worse childhood inhibitory control.

**Supplementary Information:**

The online version contains supplementary material available at 10.1186/s12940-023-01015-5.

## Introduction

Neurodevelopmental performance tasks are often separately analyzed, even when they tap into a similar construct. In particular, childhood executive functions (e.g. high-level supervisory cognitive skills vital for a child’s social and academic success (Huizinga et al. 2006; Garon et al. 2008; O’Hearn et al. 2008; Best and Miller 2010; Hughes et al. 2010; Wu et al. 2011; Diamond 2013; Lawson and Farah 2017; Simanowski and Krajewski 2019) can be challenging to precisely measure. Although many definitions of executive functions exist, recent literature identifies three core components (Diamond 2013): inhibitory control, cognitive flexibility (also known as set shifting) and working memory. Often, an executive function component is measured using a single child performance task (e.g. working memory is measured by the CANTAB spatial working memory task (Fried et al. 2015). While using a single task to tap into an executive function component can yield important information, single tasks can also be noisy indicators of executive functioning. (Willoughby, Wirth et al. 2012) This is because an executive functioning task can depend on non-executive function processes (e.g. language skills), known as task impurity. Moreover, there can be task-specific variance, in which there is error due to the specific task and not due to the underlying executive function component being measured.

To address this, latent variable approaches (Huizinga et al. 2006; Wiebe et al. 2008; Hughes et al. 2010; Willoughby et al. 2013; Karr et al. 2018) have been used to improve precision of modeling an executive function component. Rather than using a single task, a combination of tasks is used to tap into the latent construct. These chosen tasks may share little systematic non-executive function variance, allowing researchers to extract what is common among the tasks to extract a more precise measure of the executive function component. However, latent variable approaches are underused when studying the effects of risk factors on executive functions, which tends to rely on single-task analyses. (Rabin et al. 2005, 2016; Stewart et al. 2006; Chan et al. 2008; Ethier et al. 2015; Hong et al. 2015; Seo et al. 2015; Baggetta and Alexander 2016; Putnick and Bornstein 2016; Karr et al. 2018; Last et al. 2018) This may yield mixed findings for associations of an exposure-neurobehavioral outcome that can be difficult to interpret (Miyake et al. 2000; Karr et al. 2018). Separate analyses of each performance-task could also result in reduced construct validity, due to task-specific variance. Our goal was to demonstrate the utility of using item response theory (IRT) to create an integrative index of a neurodevelopmental domain, with improved measurement precision and construct validity. IRT is a set of well-established latent variable methods developed in the educational testing literature for test scoring (e.g., scoring college entrance exams), and have more recently been applied to patient reported outcomes (PRO) measurement (McHorney and Cohen 2000; Teresi et al. 2000, 2007; Chang and Reeve 2005; Dorans and Kulick 2006; Orlando Edelen et al. 2006; Perkins et al. 2006; Curran et al. 2008, Lee and Lee 2018, Aune et al. 2019), clinical assessment (Thomas 2019; Liu et al. 2020), epigenetic data (Houseman et al. 2007) and environmental exposure data (Liu et al. 2022, Chen, Feuerstahler et al. In press).

Using data from a prospective birth cohort based in Mexico City, Mexico, we derived an inhibitory control index that combines data from four inhibitory control performance-tasks. We then assessed time-varying associations of early-life lead exposure and the inhibitory control index. Lead is a neurotoxicant that has been linked to poor inhibitory control across diverse participant samples.(Surkan et al. 2007; Nicolescu et al. 2010; Plusquellec et al. 2010; Boucher et al. 2012; Ethier et al. 2015; Hong et al. 2015; Huang et al. 2016) Inhibitory control comprises of multiple components, including ability to control one’s emotions and behaviors, and ability to ignore a prompt and perform an alternate action (Carmen Usai et al. 2020). Poor inhibitory control is associated with poor academic and educational achievement, and poor mental and physical health (Diamond 2013). However, few studies have investigated the effect of time-varying lead exposure on childhood inhibitory control. Further, existing studies often assess associations between lead and each inhibitory control performance-task variable separately, which at times yields mixed findings that can be difficult to interpret (Surkan et al. 2007; Boucher et al. 2012; Ethier et al. 2015; Hong et al. 2015).

To our knowledge, this article is the first application of item response theory to create an integrative measure of inhibitory control that combines information from speed and accuracy in multiple inhibitory control tasks. We additionally developed a novel resampling approach to account for measurement error of inhibitory control factor scores in estimating associations between lead exposures and inhibitory control.

## Methods

### Population

Participants are enrolled from the Programming Research in Obesity, Growth, Environment and Social Stressors (PROGRESS) cohort, a prospective longitudinal birth cohort in Mexico City, Mexico designed to examine associations between early life environmental exposures, including lead, on neurodevelopmental outcomes in children. Detailed descriptions of recruitment, enrollment and follow-up of the cohort are available elsewhere (Braun et al. 2014; Burris et al. 2014). Of the 948 mother-child dyads initially enrolled into PROGRESS during pregnancy, between 2007 and 2011, inhibitory control task performance (at age 8–9 years) was available for 533/1052 (51%) of participants. Thus, the sample size for creating the latent variable for inhibitory control was N = 533.

Procedures were approved by institutional review boards at Harvard School of Public Health, Icahn School of Medicine at Mount Sinai, and the Mexican National Institute of Public Health. Women provided written informed consent in Spanish.

### Inhibitory control performance-tasks

We developed a measurement model for inhibitory control, using four performance tasks: Go/NoGo Happy, Go/NoGo Neutral, Go/NoGo Letter, and DKEFS Color-Word Interference Test. We used theory-driven considerations to identify the optimal indicators to represent different aspects of the performance-task (Bansal et al. 2021).

#### Go/NoGo Happy

A computerized Go/No-Go task was used, with stimuli presented using E-Prime version 3, in which participants are asked to press the spacebar as quickly as possible when a “go” stimuli was presented, and to inhibit their response when a “no-go” stimuli was presented. The “go” stimuli in the Go/No-Go Happy task was happy faces of peers, and the no-go stimuli were neutral faces of peers. From the Go/NoGo Happy task, we extracted the commission error rate (i.e., the false alarm rate), which is the rate of falsely pressing the button in no-go trials (Meule 2017). We also extracted the mean response time.

#### Go/NoGo Neutral

This task followed the same administration as the Go/No-Go Happy task, except that the “go” stimuli was neutral faces of peers, and the “no-go” stimuli was happy faces of peers. From the Go/NoGo Neutral task, we extracted the commission error rate and mean response time.

#### Go/NoGo letter

This task also followed the same administration as the Go/No-Go Happy task, except that the “go” stimuli was any letter except for “X”, and the “no-go” stimuli was the letter “X”. From the Go/NoGo Letter task, we extracted the commission error rate and mean response time.

*D-KEFS Color-Word Interference Test (CWIT) -Condition 3 (Inhibition) (*Delis, D. C., Kaplan, E., & Kramer, J. H. (2001). Delis-Kaplan executive function system: Technical manual. San Antonio, TX: The Psychological Corporation): This condition is comparable to the widely used Stroop test, in that participants are required to name the ink color, but not read the word, of color words (e.g., the word blue printed in red ink) as quickly and accurately as possible. From this task, we extracted the number of total mistakes and the completion time.

### Item response theory for inhibitory control ability

IRT is a flexible modeling approach that attempts to describe and elucidate the relations of measured items (e.g., performance-task levels) as indicators for an unobservable latent trait (e.g., latent inhibitory control ability). In an IRT framework, the probability of an individual endorsing an item (e.g., performing better on the Happy Go/NoGo task) is a function of the latent trait (e.g., inhibitory control ability) and item-specific parameters. IRT-derived scores represent estimates of the latent inhibitory control ability, and differentially weight the contributions of the individual performance-based assessments on their data-driven parameters, with a nonlinear relationship between levels of performance on individual tasks and the latent inhibitory control ability. The probability of endorsing an item increases monotonically with the level of the trait.

Using IRT, we created an integrative latent variable of inhibitory control combining four inhibitory control tasks; Go/NoGo Happy; Go/NoGo Neutral; Go/NoGo Letter; DKEFS. To create the variable, we first coded six categories which combine errors with response time. For an individual *i*, *i = 1,…n*, and task *j*, *j* = {Go/NoGo Happy; Go/NoGo Neutral; Go/NoGo Letter; DKEFS}, we used the following algorithm. For task *j*, the cutoff thresholds are sample-based. The *lower error cutoff*_*j*_ was chosen to be the score corresponding to the 25th percentile of scores on that task, and the *upper error cutoff*_*j*_ was chosen to be the score corresponding to the 75th percentile of scores on that task. The *speed cutoff*_*j*_ for the task *j* was set at the median level of scores for that task.

$${x_{i,j}} = {\rm{ }}\left\{ {\begin{array}{*{20}{l}}1,Erro{r_{i,j}} \\ \quad \ge Upper\; error\; cutof{f_j}\;and\; Spee{d_{i,j}} \\ \quad < Speed\; cutof{f_j}\\2,Erro{r_{i,j}} \\ \quad \ge Upper\; error\; cutof{f_j}\;and\; Spee{d_{i,j}}\\ \quad  \ge Speed\; cutof{f_j}\\3,Lower\; error\; cutof{f_{i,j}} \\ \quad \le Erro{r_{i,j}} \\ \quad < Upper\; error\; cutof{f_j}\;and\; Spee{d_{i,j}} \\ \quad \ge Speed\; cutof{f_j}\\4,Lower\; error\; cutof{f_{i,j}} \\ \quad \le Erro{r_{i,j}} \\ \quad < Upper\; error\; cutof{f_j}\;and\; Spee{d_{i,j}} \\ \quad < Speed\; cutof{f_j}\\5,Erro{r_{i,j}} \\ \quad < Lower\; error\; cutof{f_j}\;and\; Spee{d_{i,j}} \\ \quad \ge Speed\; cutof{f_j}\\6,Erro{r_{i,j}} \\ \quad < Lower\; error\; cutof{f_j}\;and\; Spee{d_{i,j}} \\ \quad < Speed\; cutof{f_j}\end{array}} \right.$$

These response levels for task *j* can be considered as the following: Response level 1, low accuracy and high speed, considered the poorest inhibitory control performance; Response level 6, high accuracy and high speed, considered the optimal inhibitory control performance.

$${x_{i,j}} = \left\{ {\begin{array}{*{20}{c}}{1,Low\,accuracy,high\,speed}\\{2,Low\,accuracy,low\,speed}\\{3,Mid\,accuracy,low\,speed}\\{4,Mid\,accuracy,high\,speed}\\{5,Highaccuracy,low\,speed}\\{6,High\,accuracy,high\,speed}\end{array}} \right.$$

We used generalized partial credit (GPCM) IRT models to avoid assumptions about the ordering of the middle categories. While we have imposed structure such that the 6th category being most optimal, and the 1st category as poor, we did not impose ordering on the middle categories. The GPCM assumes data-driven, nonlinear associations between the response levels of each task (“item”) and the latent inhibitory control variable. For a given task, the probability of scoring in the “high accuracy, high speed” category increases with higher inhibitory control ability. While for a given task, the probability of scoring in the low accuracy, high speed category decreases with higher inhibitory control ability. The GPCM model relates the probability of a response level *k*_*j*_ for item (task) *j*, for an individual *i*, to that individual’s latent trait of inhibitory control, ${\theta }_{i}$. The GPCM was originally developed by Muraki (1992) and details can be found there.

The latent trait (inhibitory control) is *θ*, the item (task) discrimination is *α*_*j*_ for task *j*, the *δ*_*j,k*_ corresponds to a difficulty parameter for categories of task *j*, and K is the number of categories for task *j*, *k = {1, …, K}*.

Models were fit using the R package “ltm: An R package for latent variable modelling and item response theory analyses”.(Rizopoulos 2006) We estimated an expected a posteriori estimate (EAP) for each participant, ${\widehat{\theta }}_{i}$, which is quantified as the most plausible value of the latent inhibitory control index for subject *i*, given subject *i*’s response pattern on the inhibitory control tasks and the generalized partial credit IRT model.

IRT allows each of the items (performance-tasks) to be examined graphically. The item information curve shows how well and precisely each individual task measures the latent inhibitory control trait at various levels of the latent trait. Certain items may provide more information at low levels of the latent trait, while others may provide more information at higher levels of the latent trait. The test information curve aggregates the item information curves across all the items. It tells us how well the test measures the latent trait at various levels of the latent trait. Ideally, the test information curve would peak around the mean of the sample because that is where the most individuals would be. We would also want the test information curve to be high across a range of theta values, so that we have good precision of the inhibitory control index across the range of the attribute. In IRT, because the measurement precision can differ across levels of the latent trait, inhibitory control, we also plotted the standard error of measurement across inhibitory control ($\theta$) values. We would want the standard errors to be low across a wide range of theta values.

We then tested where any tasks exhibit bias by sex, by testing for differential item functioning (DIF) (Dorans and Kulick 2006; Ramirez et al. 2006; Choi et al. 2011) by sex. Using the R package “Lordif”(Choi et al. 2011), we evaluated DIF for sex, using ordinal logistic regression models with a McFadden’s pseudo R^2 change of 2% as the critical value.(Lameijer et al. 2020) If DIF was found for a performance-task, it would suggest potential bias by score, because it suggests that if a male and a female have the same underlying level of inhibitory control, they would have different scores on the particular performance task. If DIF was detected, then it would necessitate adjustments to correct for bias by sex. Using established metrics, we tested for DIF.

The IRT model for the inhibitory control index was calibrated on N = 533 participants, who had scores on all four performance-tasks.

### Adjusted associations of lead exposure and inhibitory control

Using our inhibitory control latent variable, we used multivariable linear regression to examine associations between umbilical cord blood and 4-year lead concentrations and inhibitory control at 8–9 years of age. Venous umbilical cord blood was collected within 12 h of delivery. Blood specimens were drawn in trace metal-free tubes, refrigerated at 2–6 °C until analysis. External calibration using the Agilent 8800 ICP Triple Quad (ICP-QQQ) in MS/MS mode was used to measure the lead concentrations. The limit of detection (LOD) was < 0.2 µg/dl. All models adjusted for mother’s IQ, child sex (male/female), child age at assessment (years) and socio-economic status (SES). SES was measured at two time points (birth and 4-year follow-up). SES was measured by a validated approach developed by the Mexican Association of Marketing Research and Public Opinion Agencies (AMAI), using a weighted sum of thirteen culturally relevant indicators (e.g. education level of the head of household, type of flooring, automobiles, etc.) to define 6 levels of SES, known as the 13 × 6 rule.

We used complete case analysis to address missingness in any exposure, outcome or covariate data. The resulting sample for each model are as follows; Model 1 assessing the association between umbilical cord blood lead and inhibitory control was n = 281 (missing variables: umbilical cord blood lead (243), child age at assessment (1), maternal IQ (8). Model 2 assessing the association between 4-year blood lead and inhibitory control was n = 333 (missing variables: child lead at age 4 years (118), child age at assessment (1), maternal IQ (12) and SES at 4 years (69). Model 3 assessing the association between time-varying lead (umbilical cord blood lead and 4-year blood lead) and inhibitory control was N = 198 (missing data: umbilical cord blood or 4-year blood lead (308), child age at assessment (1), maternal IQ (4) and SES at 4 years (22).

### Sensitivity analysis to account for measurement error of inhibitory control

Because EAP scores are estimated with an error, we developed a resampling approach to account for measurement error in inhibitory control when estimating effects of lead on inhibitory control. Using plausible value imputation, we imputed 100 plausible inhibitory control factor scores for each individual. Plausible value imputation uses a multiple imputation framework that considers the estimated IRT parameters (from the GPCM) as fixed, and then estimates the plausible values of the factor scores. For each individual, we then resampled 10 plausible values with replacement, and averaged the plausible value factor scores. We treated this as a new dataset with which to estimate associations with lead. We repeated this process 100 times. Over the 100 repetitions, we made histograms of the estimated effect sizes and p-values of the regressions.

### Sensitivity analysis to adjust for exposure to secondhand smoking during 2nd trimester

We conducted additional analyses to assess the associations between pre-natal and early life lead exposure and childhood hyperactivity which also additionally adjusted for exposure to second-hand smoking during pregnancy.

### Secondary analyses of using structural equation modeling (SEM) to assess the associations

We performed additional analyses using structural equation modeling (SEM) to assess the association between pre-natal and early life lead exposure and childhood hyperactivity. We fitted 3 SEM models to examine associations between umbilical cord blood lead and inhibitory control, 4-year blood lead and inhibitory control, time-varying lead (umbilical cord blood lead and 4-year blood lead) and inhibitory control. We adjusted for the same set of covariates as what we used in the regression models using inhibitory control latent variables.

## Results

Table 1 presents the summary statistics for the overall sample. The participants (n = 533) include in the IRT inhibitory control index were 9 years of age [interquartile range (IQR: 9–10 years)], 51.4% males. Median (IQR) umbilical cord blood lead concentration (ug/dL) was 3.04 [1.65, 4.81] in males and 2.47 [1.61, 4.15] in females (p = 0.075). Median (IQR) lead concentration (ug/dL) at 4 years of age was 1.69 [1.25, 2.41] in males and 1.72 [1.31, 2.56] in females.

**Table 1:** Summary statistics for the overall sample (N = 533) with inhibitory control measures on the 4 tasks. Median and interquartile range presented. We used Mann-Whitney U test to compare continuous variables and chi-square test to compare categorical variables

	Overall	Males	Females	p-value	% Missing
n	533	274	259		
Child age in years (median [IQR])	9.00 [9.00, 10.00]	9.00 [9.00, 10.00]	9.00 [9.00, 10.00]	0.531	0.2
Umbilical cord blood lead (median [IQR])	2.76 [1.63, 4.50]	3.04 [1.65, 4.81]	2.47 [1.61, 4.15]	0.075	45.6
4-year lead (median [IQR])	1.71 [1.27, 2.51]	1.69 [1.25, 2.41]	1.72 [1.31, 2.56]	0.596	22.1
SES at birth (%)				0.589	0
1	9.9	10.2	9.7		
2	42.6	42.0	43.2		
3	23.3	22.3	24.3		
4	13.9	15.0	12.7		
5	8.8	9.9	7.7		
6	1.5	0.7	2.3		
SES at 4 years (%)				0.801	22.7
1	1.2	1.5	1.0		
2	27.2	26.1	28.2		
3	30.6	34.0	27.3		
4	21.4	20.2	22.5		
5	10.9	10.8	11.0		
6	7.5	6.4	8.6		
7	1.2	1.0	1.4		
Mother’s IQ (median [IQR])	86.00 [76.00, 94.00]	85.00 [75.00, 95.00]	86.00 [77.00, 94.00]	0.359	3.2
Happy Go/NoGo commission errors (median [IQR])	0.21 [0.12, 0.29]	0.21 [0.12, 0.33]	0.17 [0.10, 0.27]	0.018	0
Happy Go/NoGo reaction time (median [IQR])	572.01 [527.89, 617.80]	575.31 [529.36, 623.38]	567.85 [526.80, 611.08]	0.249	0
Letter Go/NoGo commission errors (median [IQR])	0.21 [0.12, 0.33]	0.21 [0.12, 0.33]	0.21 [0.12, 0.29]	0.069	0
Letter Go/NoGo reaction time (median [IQR])	498.93 [455.46, 536.86]	495.54 [454.17, 532.94]	500.62 [456.06, 542.81]	0.239	0
Neutral Go/NoGo commission errors (median [IQR])	0.21 [0.12, 0.29]	0.21 [0.12, 0.33]	0.21 [0.12, 0.29]	0.011	0
Neutral Go/NoGo reaction time (median [IQR])	572.51 [533.14, 621.40]	567.33 [529.55, 620.51]	581.01 [542.22, 622.34]	0.097	0
DKEFS condition 3 reaction time (median [IQR])	88.00 [75.00, 107.00]	87.50 [74.25, 105.00]	89.00 [76.50, 111.00]	0.133	0
DKEFS condition 3 total mistakes (median [IQR])	5.00 [3.00, 7.00]	5.00 [3.00, 8.00]	5.00 [3.00, 7.00]	0.109	0

**Fig. 1:**
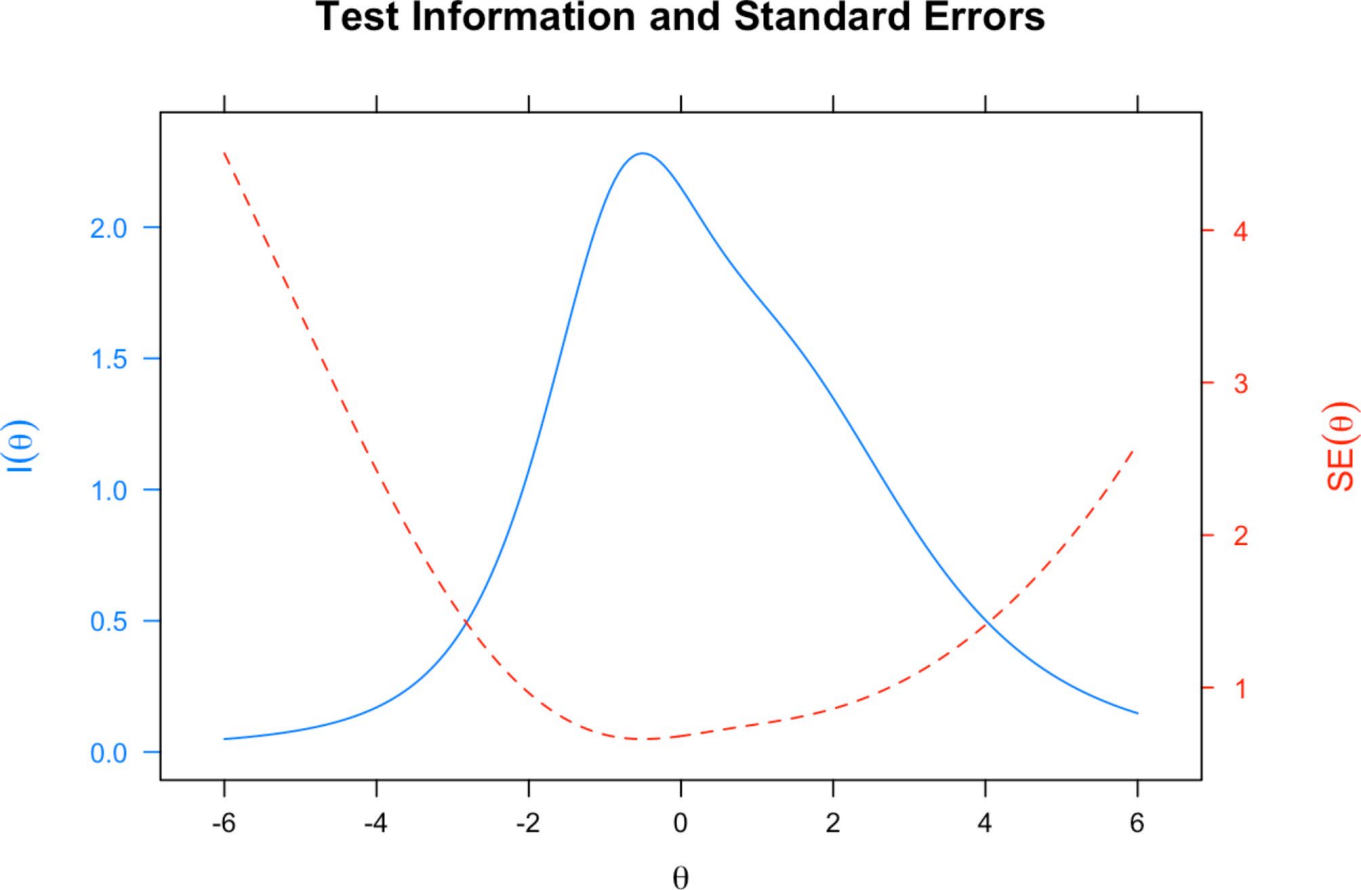
Test information curves and standard error for the overall model

Parameter estimates for the GPCM, used to calibrate the inhibitory control index, are presented in Supplementary Table 1. Item discrimination parameters ranged from 0.20 for DKEFS to 0.80 for Letter Go/NoGo, with higher discriminations indicating the task contributes more to the inhibitory control index. We did not identify DIF by child sex using our established criteria. The pseudo R^2^ changes were 0.0006, 0.0006, 0.002 and 0.0004 for Happy Go/NoGo, Letter Go/NoGo, Neutral Go/NoGo and DKEFS respectively. The test information and standard errors curves are presented in Fig. 1; the standard errors are minimized 1 SD above and below the mean.

**Fig. 2:**
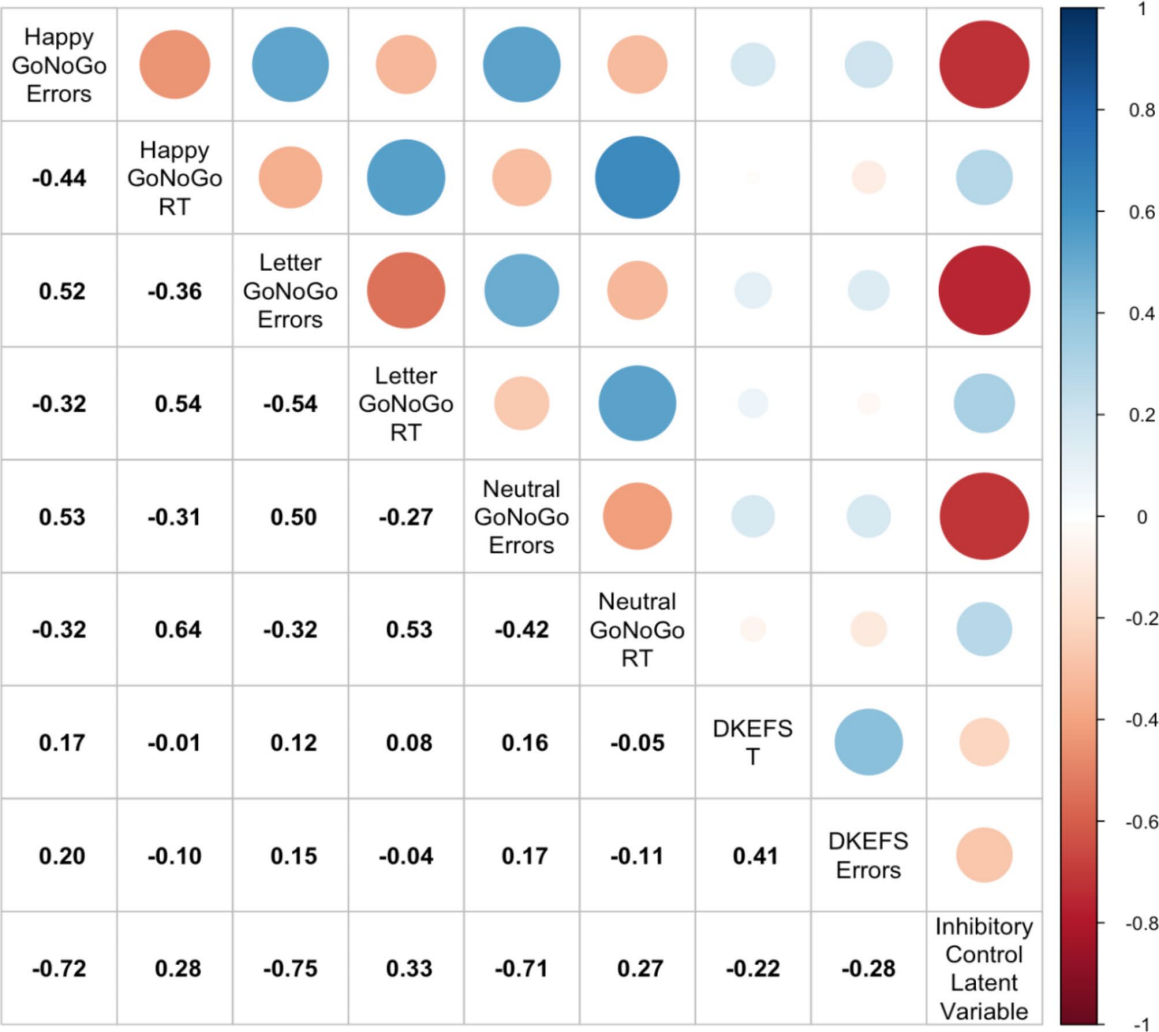
Pearson’s correlations between inhibitory control latent variable and the individual task variables

The inhibitory control index had moderate to strong negative correlations with commission errors from the three Go/NoGo tasks (Pearson’s ρ = -0.71 to -0.75), and low-moderate positive correlations with response time from the three Go/NoGo tasks (Pearson’s ρ = 0.27 to 0.33) (Fig. 2). Associations with the DKEFS total mistakes and response time were low (Pearson’s ρ = − 0.22 and ρ = -0.28, respectively).

**Fig. 3:**
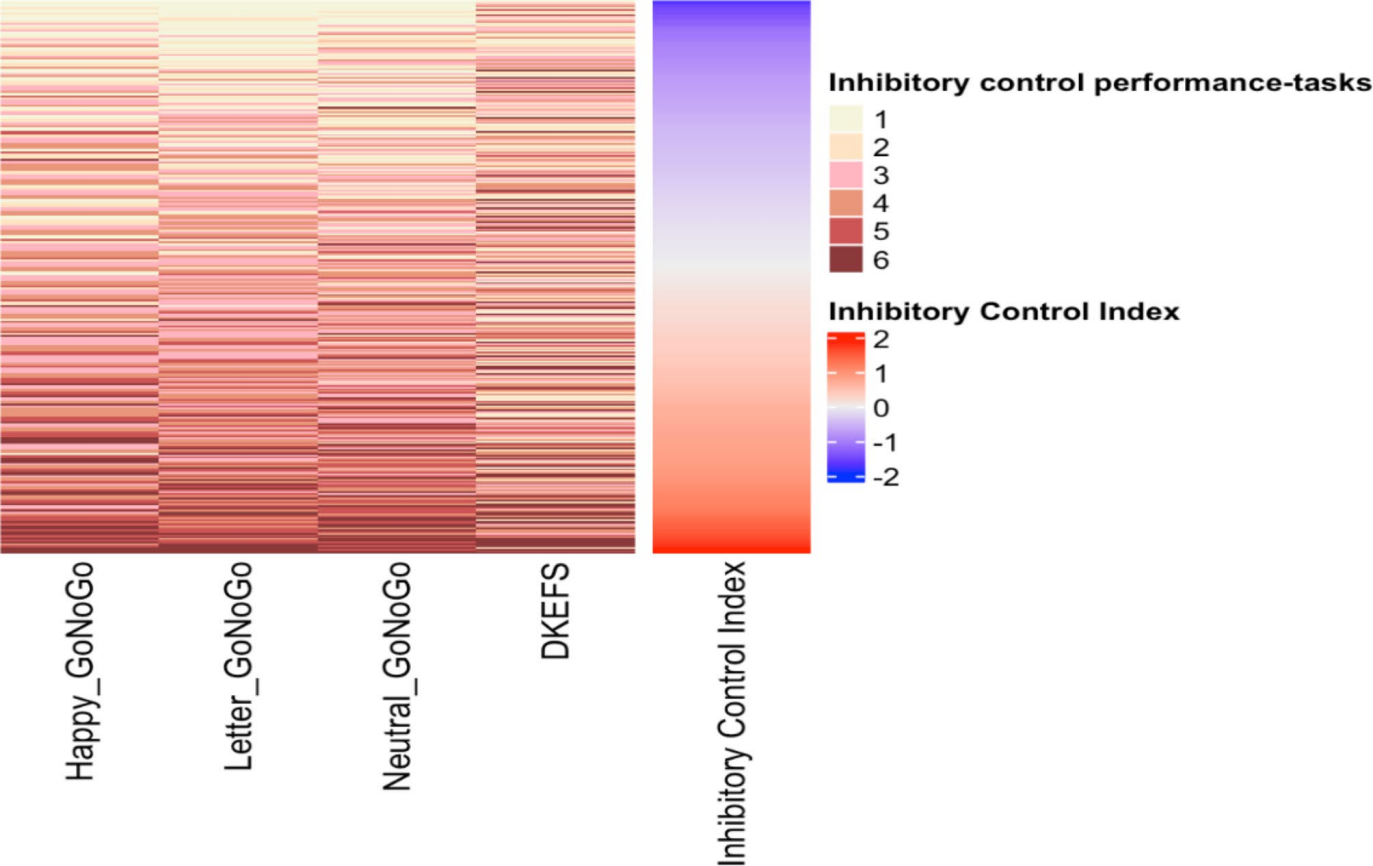
Unique response patterns on the four inhibitory control tasks in our data and the corresponding level of the inhibitory control index. (N = 318)

In Fig. 3, we present a visualization of the response patterns on the four inhibitory control tasks, and the corresponding level of the inhibitory control index. Figure 3 presents the unique response patterns in our data (N = 318), and Supplementary Fig. 1 presents the response patterns for all N = 533 participants in our data.

**Fig. 4:**
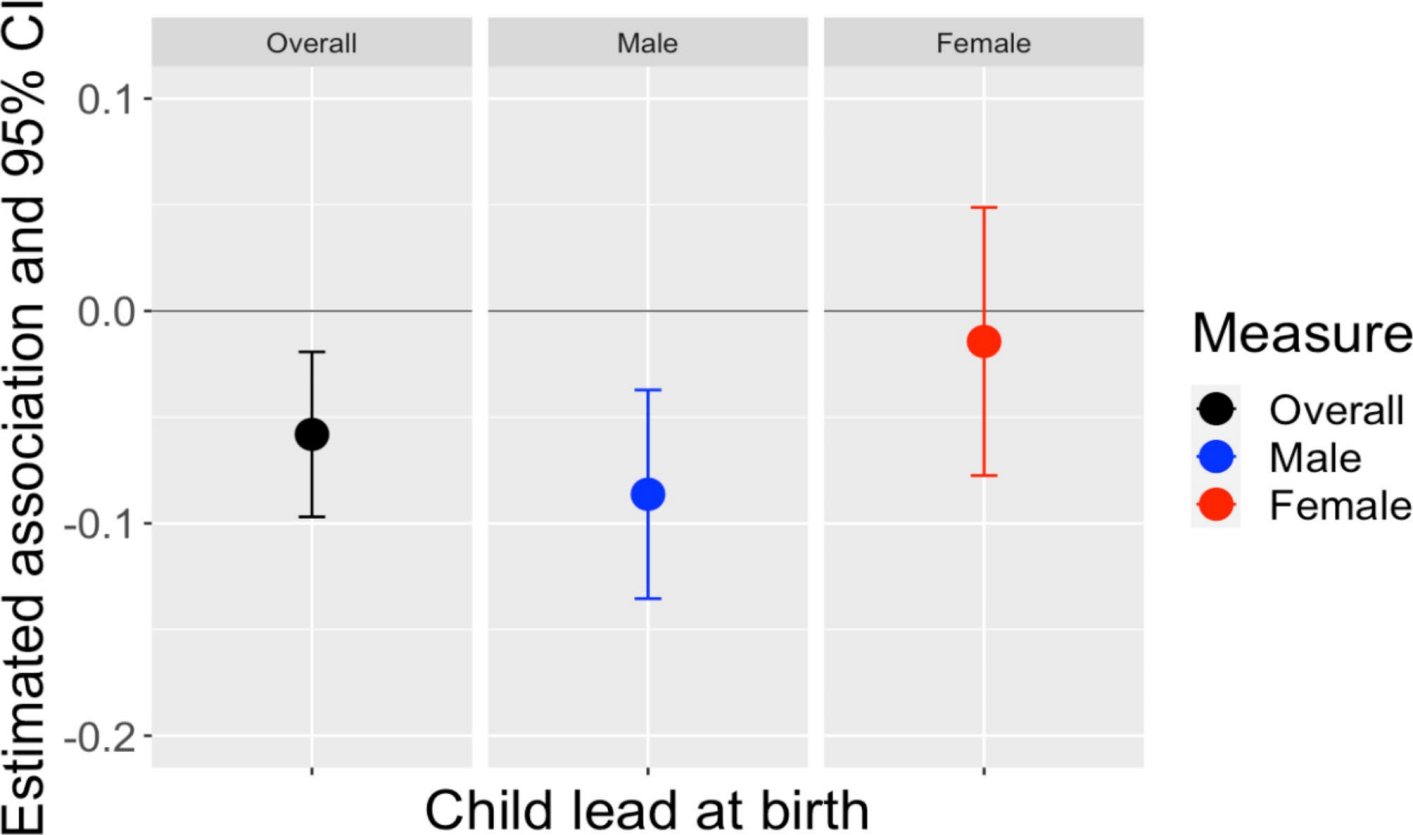
Association of umbilical cord blood and inhibitory control

We first modeled adjusted associations of umbilical cord blood lead with inhibitory control (Fig. 4, Supplementary Table 2). A 1 unit increase in lead concentrations at birth was associated with a -0.06 (95% CI: -0.10, -0.02) SD decrease in the inhibitory control index. In sex-stratified models, umbilical cord blood lead were significantly associated with lower childhood inhibitory control in males − 0.09 (95% CI: -0.14, -0.04) SD, but not in females.

**Fig. 5:**
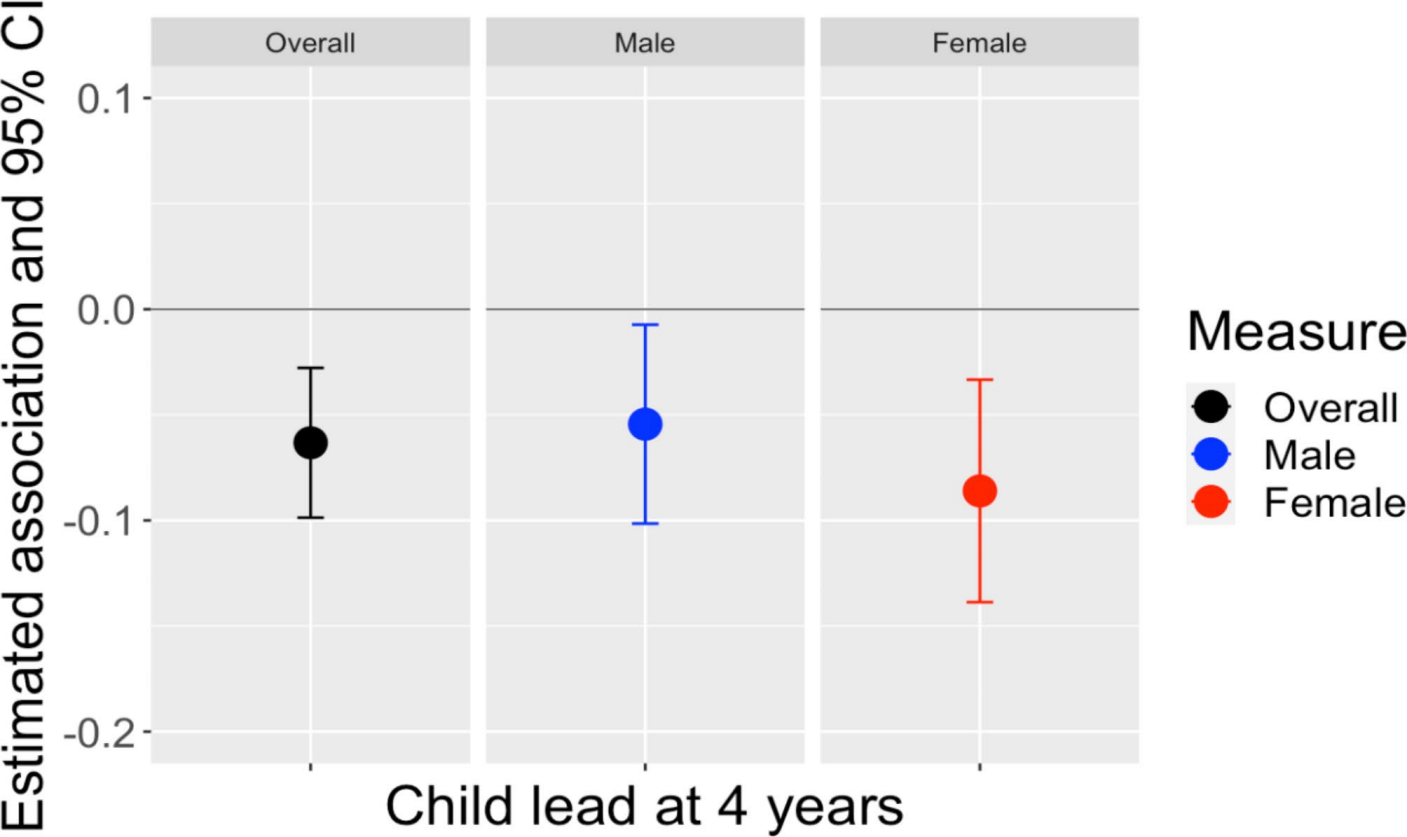
Association of lead at 4 years and inhibitory control

We then modeled adjusted associations of lead exposure at 4 years of age with childhood inhibitory control index (Fig. 5, Supplementary Table 3). A 1 unit increase in blood lead concentrations at 4 years of age was associated with a -0.06 (95% CI: -0.10, -0.03) SD decrease in the inhibitory control index. In sex-stratified models, this effect was significant for both males [-0.05 (95% CI: -0.10, -0.01)] and females [-0.09 (95% CI: -0.14, -0.03)].

**Fig. 6:**
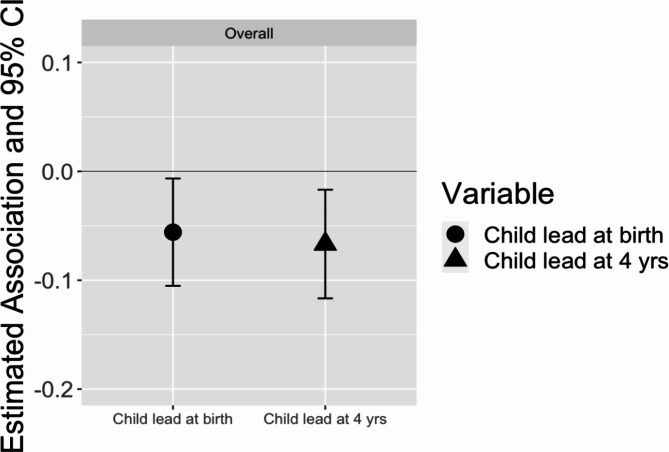
Association of time varying umbilical cord blood lead and 4-year lead and inhibitory control

Next, we modeled time-varying associations of lead exposure and childhood inhibitory control (Fig. 6, Supplementary Table 4). Both umbilical cord blood lead [-0.06 (95% CI: -0.11, -0.01)] and blood lead at 4 years of age [-0.07 (95% CI: -0.12, -0.02)] were associated with childhood inhibitory control. Due to the smaller sample size of this analysis, we did not conduct sex-stratified analyses.

Our sensitivity analysis for lead-inhibitory control associations, using a plausible value re-sampling approach to account for measurement error of the inhibitory control index, showed that prenatal (i.e., umbilical cord blood) and childhood (i.e., 4 years of age) lead exposure was associated with childhood inhibitory control (Supplementary Figs. 2, 3, 4). These sensitivity analyses are represented a boxplot graphic of the adjusted lead-inhibitory control effect sizes and p-values, estimated for different sets of plausible values of the inhibitory control index. Supplementary Fig. 4 shows that for time-varying adjusted associations of lead and inhibitory control, when measurement error of the inhibitory control index is accounted for, lead exposure at 4 years of age is consistently associated with childhood inhibitory control, but umbilical cord blood lead is only sometimes found to be associated with childhood inhibitory control.

Lastly, we conducted a secondary analysis in which we modeled time-varying associations of lead exposure with each inhibitory control performance-task variable separately (8 models; each task has two variables for errors and response time). Supplementary Table 5 shows the adjusted effect sizes for these models. Umbilical cord blood lead was significantly associated with 3 out of 8 measures (more Happy Go/NoGo commission errors, more Neutral Go/NoGo commission errors, short Neutral Go/NoGo reaction time. 4-year lead was significantly associated with 3 out of 8 measures (more Happy Go/NoGo commission errors, more Letter Go/NoGo commission errors, and more DKEFS condition 3 total mistakes).

Findings were consistent after additional adjustment for secondhand smoke exposure during pregnancy (Supplementary Table 6).

Supplementary Fig. 5 shows the 3 SEM models: Model 1 (Supplementary Fig. 5A) assess the association between umbilical cord blood lead and inhibitory control, adjusting for mother’s IQ, SES at birth, child sex and child age at assessment; Model 2 (Supplementary Fig. 5B) assess the association between 4-year blood lead and inhibitory control, adjusting for SES at 4 years, child sex and child age at assessment; Model 2 (Supplementary Fig. 5C) assess the association between time-varying lead (umbilical cord blood lead and 4-year blood lead) and inhibitory control, adjusting for mother’s IQ, SES at birth and at 4 years, child sex and child age at assessment. Fit statistics of these models are presented in Supplementary Table 7. We found significant negative association between umbilical cord blood lead and childhood hyperactivity both in Model 1 (β = -0.092, p = 0.024) and in Model 3 (β = -0.117, p = 0.009). However, we didn’t find significant association between 4-year blood lead and childhood inhibitory control using SEM models. (Supplementary Table 8)

## Discussion

We analyzed data from a prospective birth cohort in Mexico, in order to develop an integrative index of inhibitory control using item response theory to combine information about speed and accuracy across four performance tasks. Prenatal and childhood blood were independently associated with poorer childhood inhibitory control. Our sensitivity analyses, which accounted for measurement error of the inhibitory control index, suggested that childhood blood lead at 4 years of age was consistently associated with poorer childhood inhibitory control, but child lead at birth was only sometimes associated. To our knowledge, this is the first application of IRT to create an integrative measure of inhibitory control that combines information from speed and accuracy in multiple inhibitory control tasks. We additionally developed a novel resampling approach to account for measurement error of inhibitory control factor scores in estimating associations between lead exposures and inhibitory control.

Pre and post-natal lead exposure has been linked to poorer inhibitory control in multiple studies across diverse childhood populations.(Surkan et al. 2007; Nicolescu et al. 2010; Plusquellec et al. 2010; Boucher et al. 2012; Ethier et al. 2015; Hong et al. 2015; Huang et al. 2016) However, these studies have often conducted separate analyses for each task variable. While they may extract more than one variable from the same task, they separately assessed associations between lead and each task variable, sometimes yielding mixed findings which could be challenging to interpret. For example, Boucher et al. found that postnatal lead exposure was associated with accuracy of Go/NoGo tasks, but not with mean response time in a population of Inuit children (mean age 11 years).(Boucher et al. 2012) Hong et al. found that blood lead was significantly associated with commission errors, but not with response time variability of a computerized Continuous Performance Test (CPT) in South Korean children.(Hong et al. 2015) Meanwhile, Ethier et al. found that maternal blood lead was associated with greater false alarm, but not associated with reaction time, omission error or accuracy of a modified Posner paradigm for children of Arctic Quebec.(Ethier et al. 2015) Surkan et al. found associations between lead and fewer number of categories achieved and more preservation errors, but did not find associations between blood lead and SWCT scores in urban and rural US children.(Surkan et al. 2007) Our IRT approach provides a novel way to combine speed and accuracy information using a theorized framework, as low accuracy combined with fast response time is conceptualized to be indicative of poor inhibitory control.

An IRT approach, based on a theoretical neurodevelopmental framework, could be used in other epidemiological studies to integrate neuropsychological task data for increased measurement precision and construct validity, and account for measurement error. Examination of associations of other risk factors with neurodevelopment using this approach may yield new findings for prevention and intervention. Here, we demonstrated an application of IRT by calculating an overall index of inhibitory control. Many potential extensions for IRT exist, including accounting for correlated, multi-dimensional constructs (for example, multi-dimensional IRT or bi-factor models could be used to calculate overall and construct-specific scores for correlated cognitive constructs and integrate data from multiple tasks. IRT could enable researchers studying executive functioning across fields (e.g., environmental health, pediatric neuropsychology) to use data-driven approaches that reduce the dimensionality of their data and thereby concerns about multiple comparisons.

We also used SEM method and compared with the IRT approach. We found significant association between umbilical cord blood lead and childhood hyperactivity in SEM models, which was consistent with our findings using the IRT approach. However, we didn’t find significant association between 4-year blood lead and inhibitory control using SEM, while we found significant associations using the IRT approach. The IRT approach may be more sensitive to detect associations in this study.

## Electronic supplementary material

Below is the link to the electronic supplementary material.

Supplementary Material 1

## Data Availability

Data and code will be made available upon reasonable request to the corresponding author.
