# Supplementary material for: Pre-natal and early life lead exposure and childhood inhibitory control: an item response theory approach to improve measurement precision of inhibitory control
Source: Environ Health. 2024 Sep 5;23:71. doi: 10.1186/s12940-023-01015-5 (PMC11375946; doi:10.1186/s12940-023-01015-5)
Supplement: Supplementary file 1 — Supplementary Material 1 [file 12940_2023_1015_MOESM1_ESM.docx]

Pre-natal and early life lead exposure and childhood inhibitory control: An item response theory approach to improve measurement precision of inhibitory control

Shelley H. Liu, PhD^1^, Yitong Chen, MS^1^, David Bellinger, PhD^2^, Erik de Water, PhD^3^, Megan Horton, PhD^4^, Martha M Téllez-Rojo, PhD^4^, Robert Wright, MD^5^

^1^Department of Population Health Science and Policy, Icahn School of Medicine at Mount Sinai, New York, NY, USA

^2^Department of Neurology, Boston Children’s Hospital, Boston, MA, USA

^3^Great Lakes Neurobehavioral Center, Edina, MN, USA

^4^Department of Environmental Medicine and Public Health, Icahn School of Medicine at Mount Sinai

^5^Center for Nutrition and Health Research, National Institute of Public Health, Mexico

**Supplementary Table 1:** Parameter estimates from the generalized partial credit model to create an integrative index for the four inhibitory control tasks.

| **Task** | **a1** | **b1** | **b2** | **b3** | **b4** | **b5** |
| --- | --- | --- | --- | --- | --- | --- |
| Happy GoNoGo | 0.653 | 0.509 | -2.006 | 0.297 | 1.292 | 2.108 |
| Letter GoNoGo | 0.801 | 0.827 | -2.352 | 0.347 | 1.278 | 2.632 |
| Neutral GoNoGo | 0.600 | 0.537 | -2.376 | 0.244 | 1.585 | 2.061 |
| DKEFS | 0.200 | -4.509 | -0.357 | 0.032 | 7.941 | -4.828 |

**Supplementary Figure 1:** Heatmap of the response patterns on the four inhibitory control tasks for all participants (N=533) and the corresponding level of the inhibitory control index.


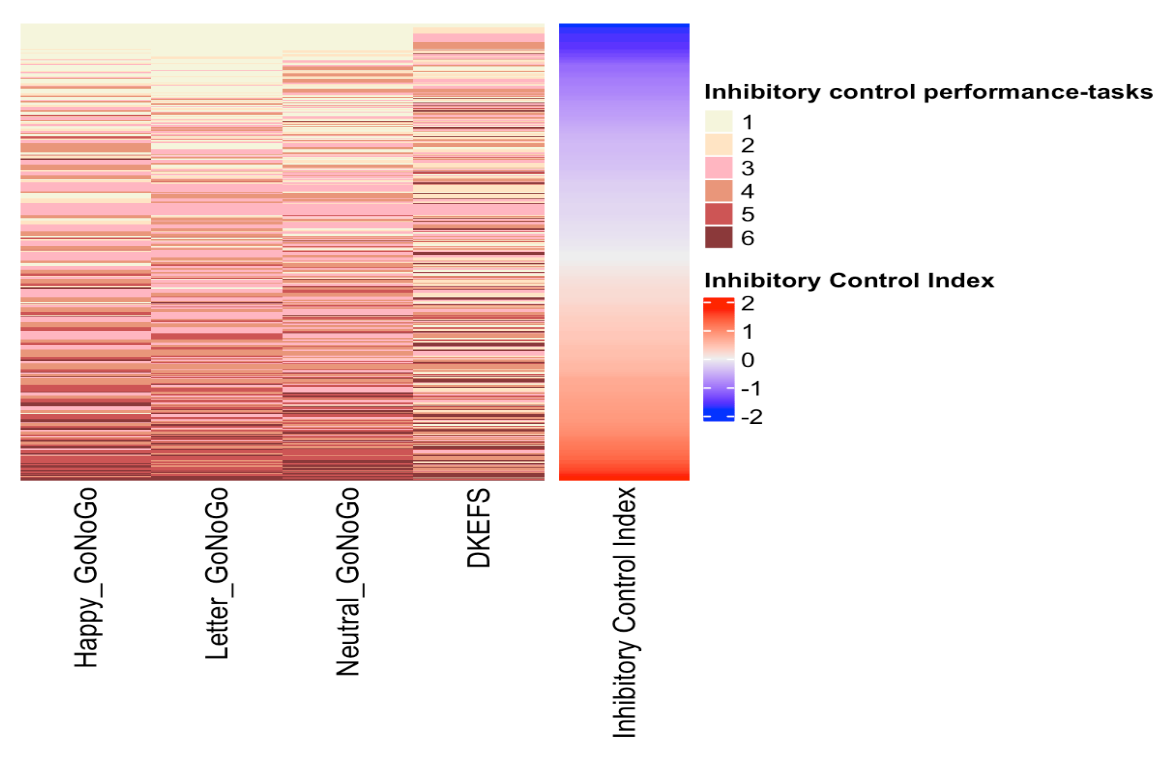


**Supplementary Table 2:** Adjusted associations of blood lead at birth and childhood inhibitory control index for the overall sample, and for sex-stratified models.

|  | **Overall** | | | **Male** | | | **Female** | | |
| --- | --- | --- | --- | --- | --- | --- | --- | --- | --- |
| *Predictors* | *Estimates* | *CI* | *p* | *Estimates* | *CI* | *p* | *Estimates* | *CI* | *p* |
| (Intercept) | -2.15 | -3.57 – -0.72 | **0.003** | -1.55 | -3.44 – 0.35 | 0.109 | -2.83 | -5.10 – -0.57 | **0.015** |
| Mother’s IQ | 0.00 | -0.01 – 0.01 | 0.572 | -0.00 | -0.01 – 0.01 | 0.864 | 0.00 | -0.01 – 0.02 | 0.577 |
| Child blood lead at birth | -0.06 | -0.10 – -0.02 | **0.004** | -0.09 | -0.14 – -0.04 | **0.001** | -0.01 | -0.08 – 0.05 | 0.656 |
| SES at birth |  |  |  |  |  |  |  |  |  |
| 1 | Ref |  |  |  |  |  |  |  |  |
| 2 | -0.14 | -0.46 – 0.18 | 0.404 | 0.15 | -0.27 – 0.58 | 0.468 | -0.51 | -1.01 – 0.00 | 0.051 |
| 3 | -0.14 | -0.48 – 0.20 | 0.431 | 0.18 | -0.28 – 0.64 | 0.435 | -0.49 | -1.01 – 0.04 | 0.069 |
| 4 | 0.06 | -0.33 – 0.45 | 0.759 | 0.29 | -0.23 – 0.81 | 0.266 | -0.17 | -0.78 – 0.43 | 0.578 |
| 5 | 0.00 | -0.43 – 0.44 | 0.989 | 0.26 | -0.31 – 0.83 | 0.364 | -0.25 | -0.93 – 0.43 | 0.470 |
| 6 | -0.68 | -1.42 – 0.06 | 0.071 | -0.02 | -1.55 – 1.51 | 0.977 | -0.98 | -1.89 – -0.06 | **0.037** |
| Child sex |  |  |  |  |  |  |  |  |  |
| Male | Ref |  |  |  |  |  |  |  |  |
| Female | 0.18 | 0.00 – 0.37 | **0.048** |  |  |  |  |  |  |
| Child age at assessment | 0.23 | 0.10 – 0.36 | **0.001** | 0.17 | 0.00 – 0.34 | **0.048** | 0.33 | 0.13 – 0.53 | **0.002** |
| Observations | 281 | | | 156 | | | 125 | | |

**Supplementary Table 3.** Adjusted associations of lead exposure at 4 years and inhibitory control index, for the overall sample, and for sex-stratified models.

|  | **Overall** | | | **Male** | | | **Female** | | |
| --- | --- | --- | --- | --- | --- | --- | --- | --- | --- |
| *Predictors* | *Estimates* | *CI* | *p* | *Estimates* | *CI* | *p* | *Estimates* | *CI* | *p* |
| (Intercept) | -2.40 | -3.97 – -0.83 | **0.003** | -2.79 | -5.06 – -0.52 | **0.016** | -1.90 | -4.05 – 0.26 | 0.084 |
| Mother’s IQ | 0.00 | -0.00 – 0.01 | 0.398 | 0.01 | -0.00 – 0.02 | 0.050 | -0.00 | -0.01 – 0.01 | 0.566 |
| Child lead at 4 years | -0.06 | -0.10 – -0.03 | **0.001** | -0.05 | -0.10 – -0.01 | **0.025** | -0.09 | -0.14 – -0.03 | **0.002** |
| SES at 4 years |  |  |  |  |  |  |  |  |  |
| 1 | Ref |  |  |  |  |  |  |  |  |
| 2 | -0.08 | -0.88 – 0.71 | 0.834 | 0.59 | -0.52 – 1.69 | 0.295 | -0.54 | -1.68 – 0.60 | 0.352 |
| 3 | -0.15 | -0.94 – 0.64 | 0.705 | 0.42 | -0.66 – 1.51 | 0.443 | -0.53 | -1.68 – 0.61 | 0.358 |
| 4 | 0.00 | -0.79 – 0.80 | 0.993 | 0.53 | -0.57 – 1.62 | 0.342 | -0.32 | -1.47 – 0.83 | 0.582 |
| 5 | -0.38 | -1.20 – 0.43 | 0.359 | 0.11 | -1.01 – 1.22 | 0.852 | -0.74 | -1.91 – 0.44 | 0.219 |
| 6 | -0.21 | -1.06 – 0.64 | 0.632 | 0.99 | -0.20 – 2.17 | 0.103 | -1.04 | -2.26 – 0.18 | 0.095 |
| 7 | -0.33 | -1.67 – 1.02 | 0.634 | 0.13 | -1.74 – 1.99 | 0.891 | -0.34 | -2.29 – 1.61 | 0.731 |
| Child sex |  |  |  |  |  |  |  |  |  |
| Male | Ref |  |  |  |  |  |  |  |  |
| Female | 0.29 | 0.12 – 0.46 | **0.001** |  |  |  |  |  |  |
| Child age at assessment | 0.24 | 0.11 – 0.37 | **<0.001** | 0.15 | -0.03 – 0.34 | 0.103 | 0.33 | 0.15 – 0.51 | **<0.001** |
| Observations | 333 | | | 163 | | | 170 | | |

**Supplementary Table 4.** Adjusted time-varying associations of lead and inhibitory control index, for the overall sample

|  | **Overall** | | |
| --- | --- | --- | --- |
| *Predictors* | *Estimates* | *CI* | *p* |
| (Intercept) | -2.72 | -4.70 – -0.74 | **0.007** |
| Mother’s IQ | 0.01 | -0.00 – 0.02 | 0.148 |
| Child blood lead at birth | -0.06 | -0.11 – -0.01 | **0.028** |
| Child lead at 4 years | -0.07 | -0.12 – -0.02 | **0.010** |
| SES at birth |  |  |  |
| 1 | Ref |  |  |
| 2 | -0.13 | -0.53 – 0.26 | 0.509 |
| 3 | -0.11 | -0.53 – 0.31 | 0.603 |
| 4 | 0.01 | -0.48 – 0.49 | 0.975 |
| 5 | 0.03 | -0.51 – 0.57 | 0.907 |
| 6 | -0.73 | -1.69 – 0.23 | 0.137 |
| SES at 4 years |  |  |  |
| 1 |  |  |  |
| 2 | -0.13 | -1.05 – 0.79 | 0.780 |
| 3 | -0.22 | -1.14 – 0.69 | 0.632 |
| 4 | 0.05 | -0.89 – 0.98 | 0.924 |
| 5 | -0.47 | -1.42 – 0.48 | 0.328 |
| 6 | -0.23 | -1.27 – 0.81 | 0.659 |
| 7 | -0.61 | -2.41 – 1.19 | 0.506 |
| Child sex |  |  |  |
| Male | Ref |  |  |
| Female | 0.21 | -0.01 – 0.44 | 0.066 |
| Child age at assessment | 0.28 | 0.12 – 0.44 | **0.001** |
| Observations | 198 | | |

**Supplementary Figure 2:** Sensitivity analysis for the association of blood lead at birth and childhood inhibitory control index. Because the inhibitory control index is estimated with error, we conducted a sensitivity analysis to confirm that our lead-inhibitory control associations are still significant when we account for the error in the inhibitory control index. We used plausible value imputation to impute plausible values of the inhibitory control index for each participant, and then re-estimated the adjusted lead-inhibitory control index associations using the plausible values of the inhibitory control index. We repeated this process 100 times. In all 100 out of 100 repetitions, lead concentrations at birth were significantly associated with inhibitory control at the alpha = 0.05 significance level for the overall sample. In sex-stratified models, birth lead-inhibitory control associations were significant for males in 100 out of 100 repetitions, and non-significant for females in 100 out of 100 repetitions. The boxplots of the estimated effect sizes and p-values are presented below for the repetitions using plausible values of the inhibitory control index. The yellow dot indicates the effect size or p-value corresponding to the actual values of our inhibitory control index (the EAP scores).


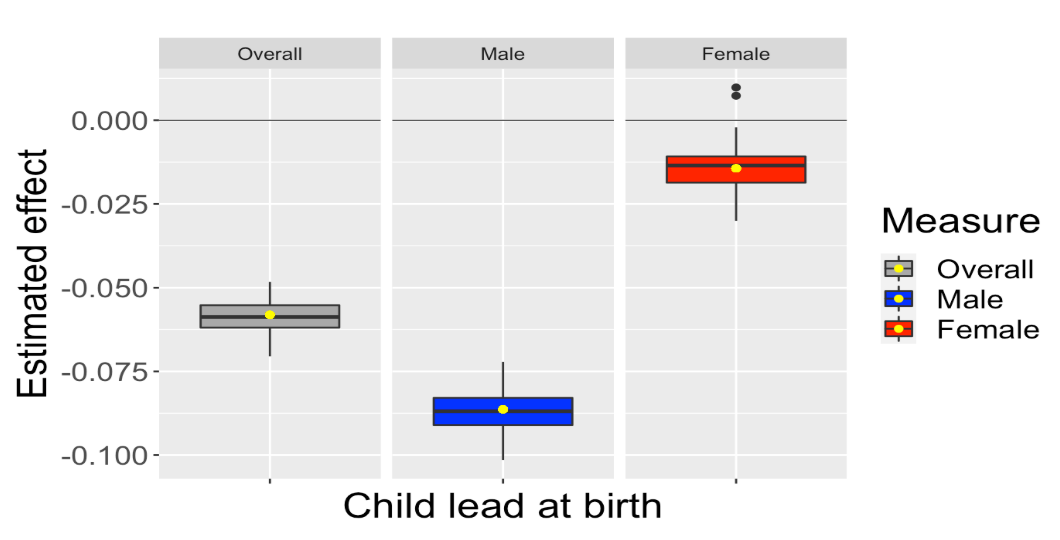


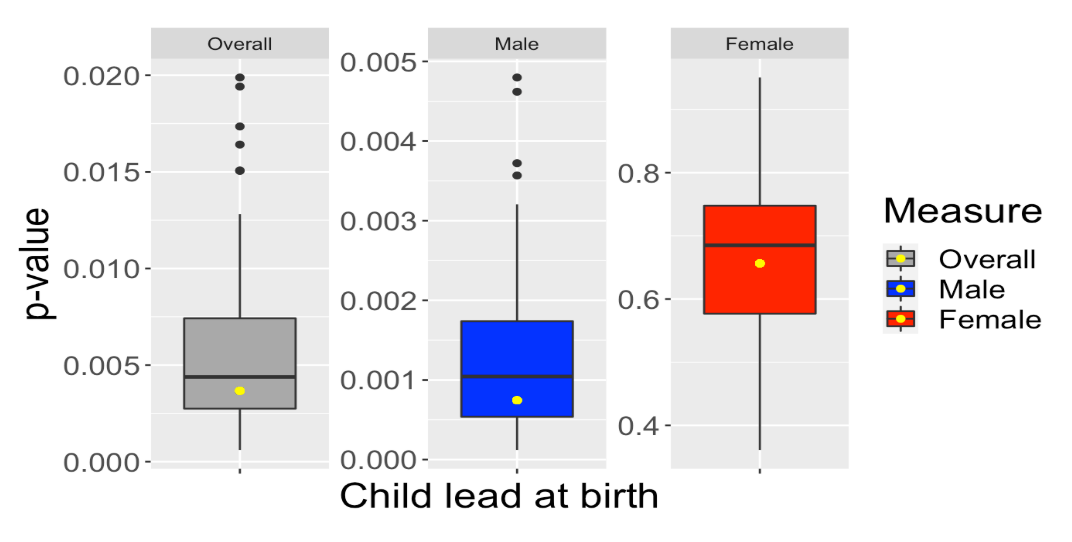


**Supplementary Figure 3:** Sensitivity analysis for the association of blood lead at 4 years of age and childhood inhibitory control.Significant associations were found in all 100 times resampling for overall and female, and significant associations were found in 90 of the 100 times resampling for male.


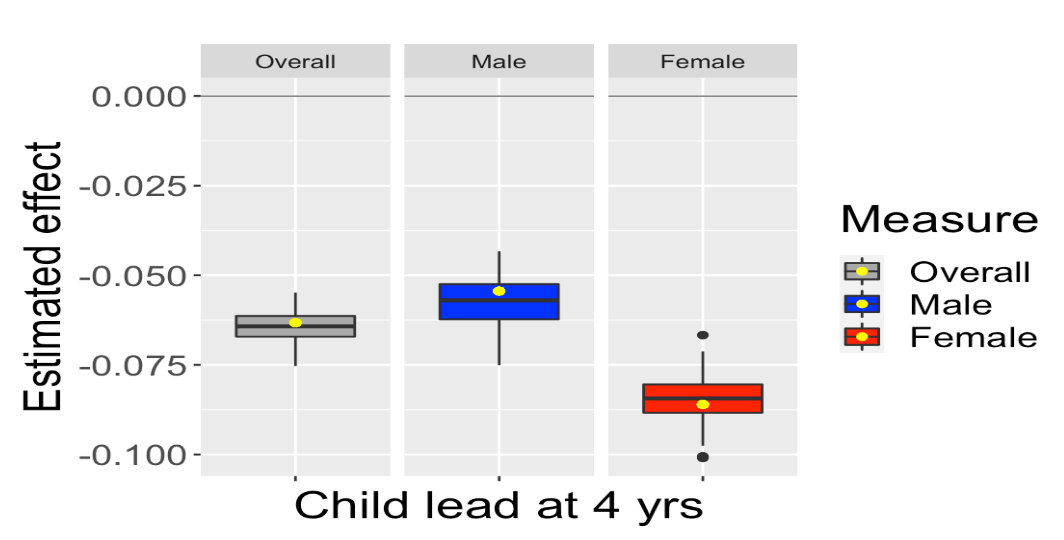


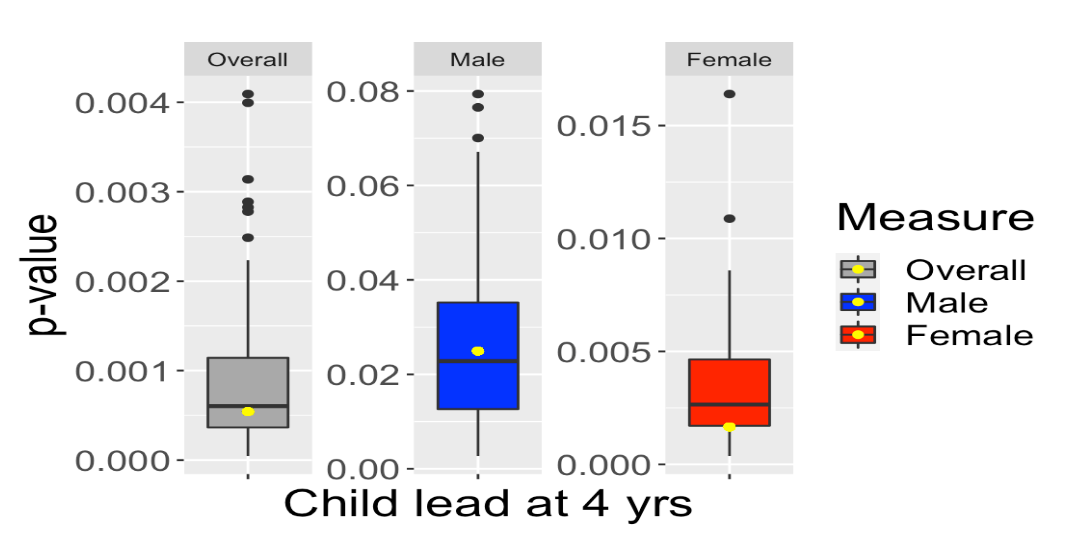


**Supplementary Figure 4:** Sensitivity analysis for the adjusted association of time-varying blood lead (birth, 4 years of age) and childhood inhibitory control. We found significant association between child lead at birth and inhibitory control in 81 of the 100 times resampling, and significant association between child lead at 4 years and inhibitory control in all 100 times resampling.


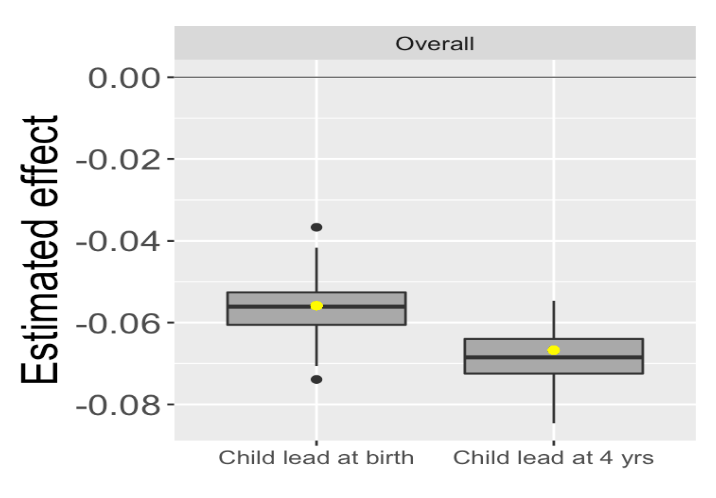

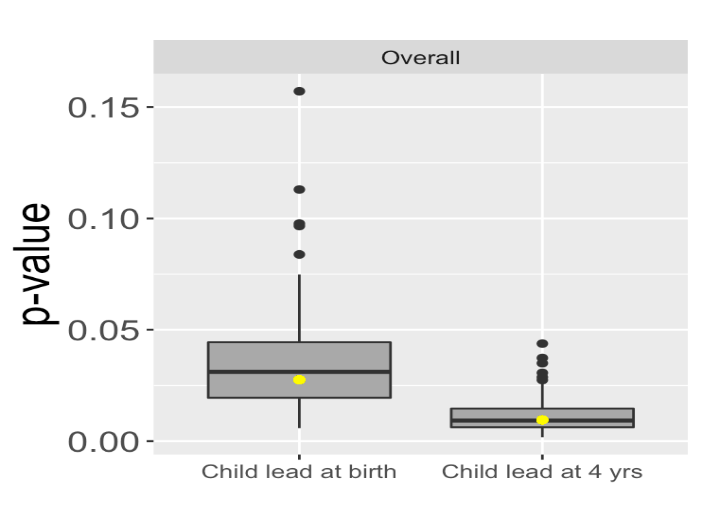


**Supplementary Table 5:** Adjusted effect size of lead and each individual task.

|  | Estimate | P value |
| --- | --- | --- |
| Happy Go/NoGo reaction time | | |
| Umbilical cord blood lead | 1.02 | 0.674 |
| 4-year lead | -3.798 | 0.123 |
| Happy Go/NoGo commission errors | | |
| Umbilical cord blood lead | 0.01 | 0.039 |
| 4-year lead | 0.01 | 0.042 |
| Letter Go/NoGo reaction time | | |
| Umbilical cord blood lead | -1.038 | 0.637 |
| 4-year lead | -2.294 | 0.304 |
| Letter Go/NoGo commission errors | | |
| Umbilical cord blood lead | 0.006 | 0.241 |
| 4-year lead | 0.017 | 0.001 |
| Neutral Go/NoGo reaction time | | |
| Umbilical cord blood lead | -5.286 | 0.048 |
| 4-year lead | -4.317 | 0.111 |
| Neutral Go/NoGo commission errors | | |
| Umbilical cord blood lead | 0.017 | 0.001 |
| 4-year lead | 0.004 | 0.371 |
| DKEFS condition 3 reaction time | | |
| Umbilical cord blood lead | -0.652 | 0.396 |
| 4-year lead | 0.755 | 0.332 |
| DKEFS condition 3 total mistakes | | |
| Umbilical cord blood lead | 0.008 | 0.949 |
| 4-year lead | 0.307 | 0.014 |

**Supplementary Table 6:** Adjusted associations of blood lead at birth and inhibitory control index, lead exposure at 4 years and inhibitory control index, and time-varying associations of lead and inhibitory control index which also adjusting for exposure to secondhand smoking in the sensitivity analyses.

| Model | Lead exposure | Adjusted association (95% CI) | p-value |
| --- | --- | --- | --- |
| At birth | Cord blood lead | -0.06 [-0.10, -0.02] | 0.004 |
| At 4 years | Blood lead at 4 years | -0.06 [-0.10, -0.03] | 0.001 |
| Time-varying model | Cord blood lead | -0.06 [-0.11, -0.01] | 0.022 |
|  | Blood lead at 4 years | -0.06 [-0.11, -0.01] | 0.013 |

**Supplementary Figure 5:** Structural equation models of blood lead at birth and childhood inhibitory control, blood lead at 4 years of age and childhood inhibitory control, time-varying blood lead (birth, 4 years of age) and childhood inhibitory control.

Supplementary Figure 5A: SEM model at birth


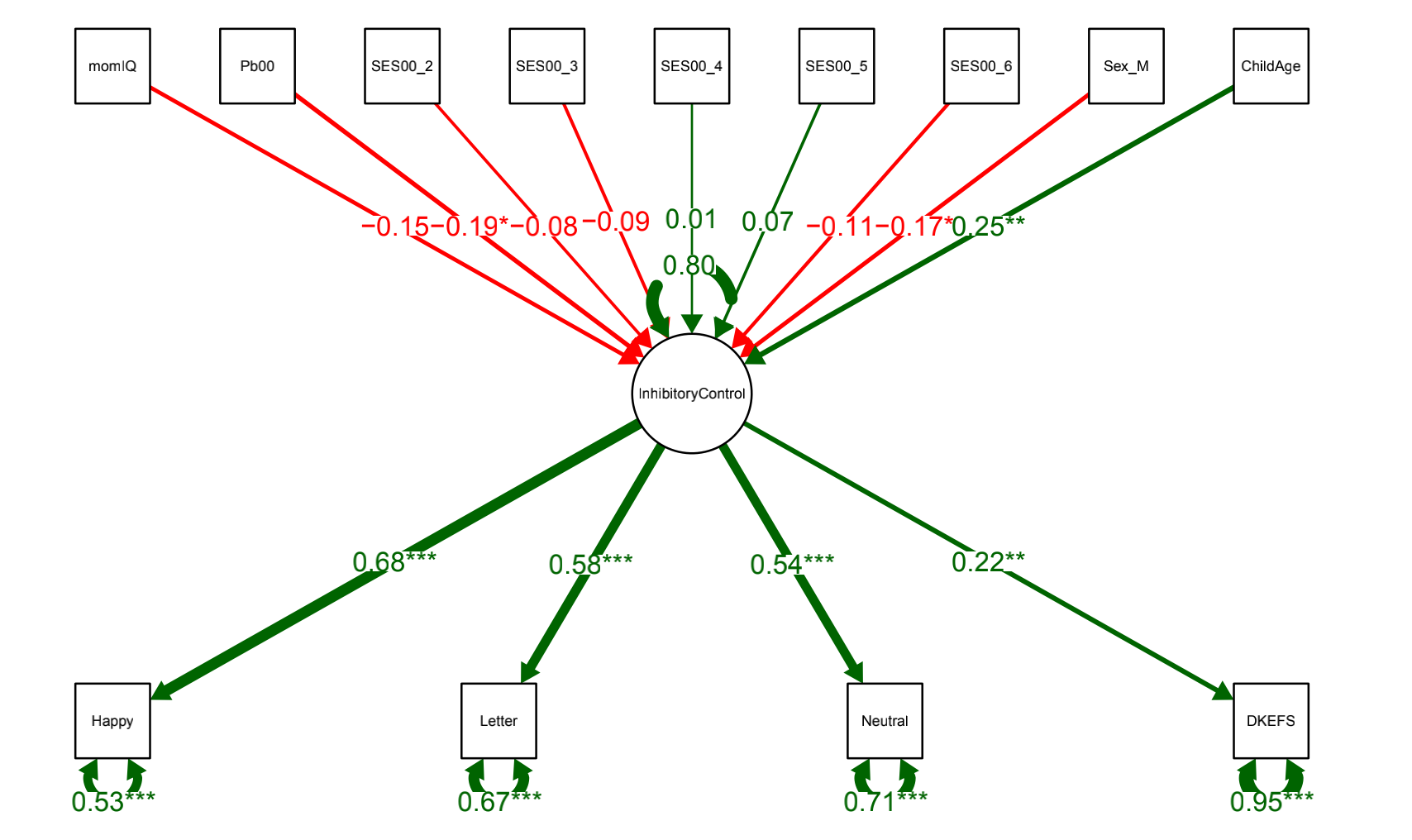


Supplementary Figure 5B: SEM model at 4 years


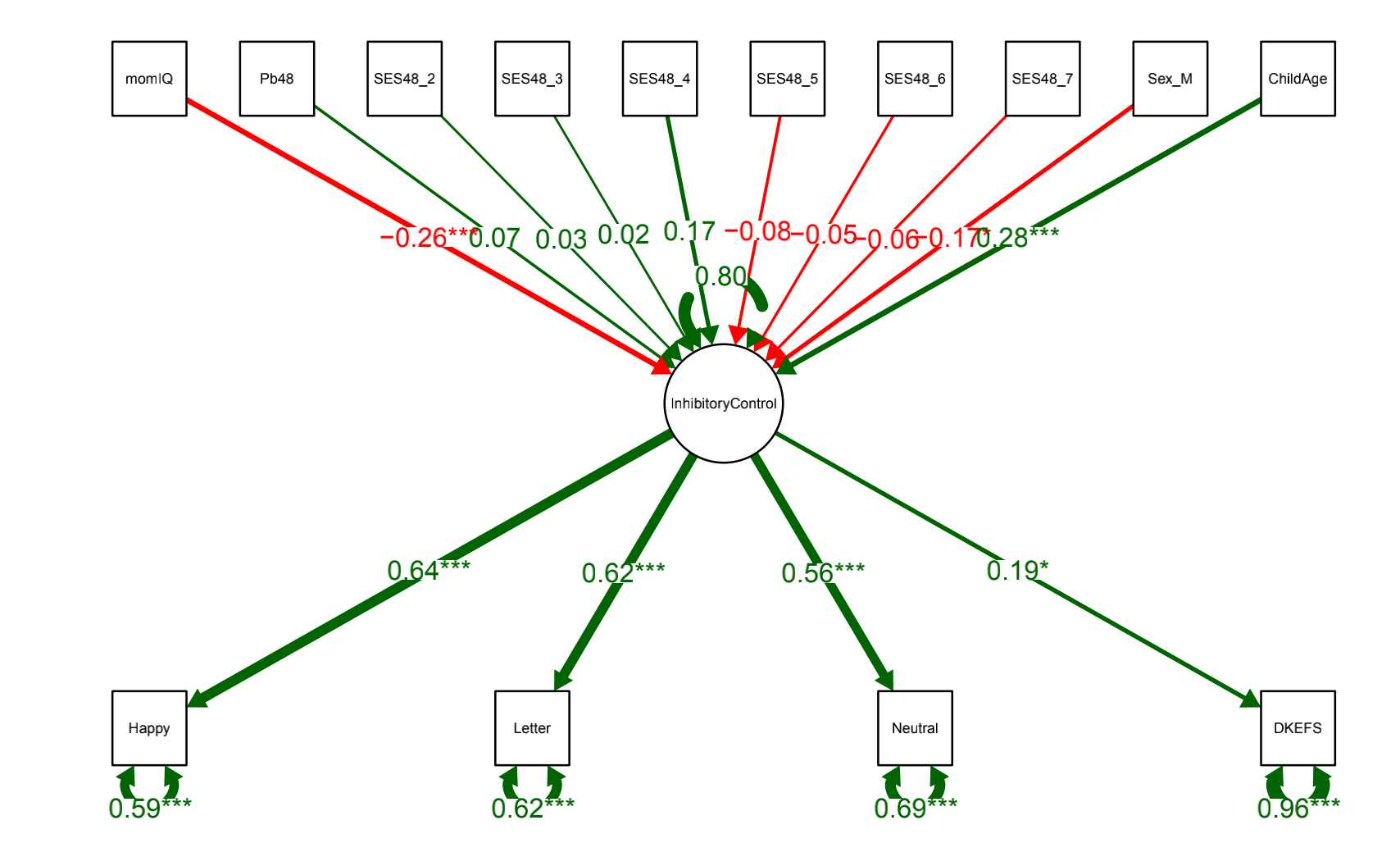


Supplementary Figure 5C: Time-varying SEM model


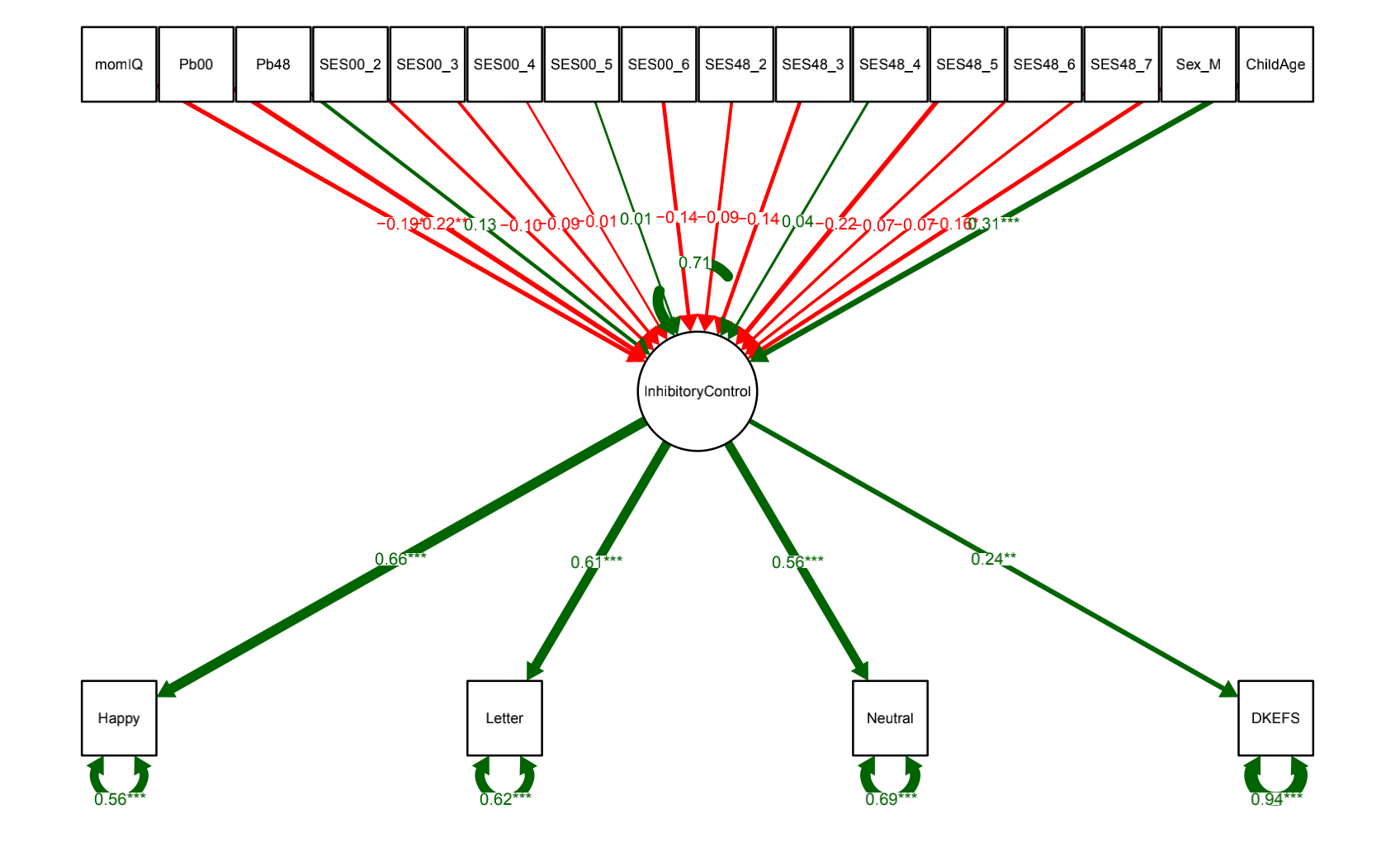


**Supplementary Table 7:** Fit statistics of the SEM models

| Model | Χ^2^ (df, p) | NFI | CFI | RMSEA (RMSEA CI) | SRMR |
| --- | --- | --- | --- | --- | --- |
| At birth | 26.650 (29, 0.591) | 0.814 | 1.000 | 0.00 (0.00, 0.05) | 0.031 |
| At 4 years | 49.364 (32, 0.026) | 0.716 | 0.864 | 0.05 (0.02, 0.07) | 0.039 |
| Time-varying | 59.168 (50, 0.176) | 0.679 | 0.920 | 0.03 (0.00, 0.06) | 0.032 |

**Supplementary Table 8:** Estimated association of blood lead and inhibitory control. Effect sizes were adjusted for mother’s IQ, SES at birth, child sex and child age at assessment in the model at birth; adjusted for mother’s IQ, SES at 4 years, child sex and child age at assessment in the model at 4 years of age; and for mother’s IQ, SES at birth and at 4 years, child sex and child age at assessment in the time varying model.

| Model | Lead exposure | Estimate | p-value |
| --- | --- | --- | --- |
| At birth | Cord blood lead | -0.092 | 0.024 |
| At 4 years | Blood lead at 4 years | 0.006 | 0.381 |
| Time-varying model | Cord blood lead | -0.117 | 0.009 |
|  | Blood lead at 4 years | 0.012 | 0.149 |
